# Délais d'entrée aux soins des personnes vivant avec le VIH dans deux centres de traitement ambulatoire de Libreville, Gabon, entre 2012 et 2020

**DOI:** 10.48327/mtsi.v5i1.2025.537

**Published:** 2025-02-11

**Authors:** Michèle Marion NTSAME OWONO, Magalie ESSOMEYO NGUE MEBALE, Charleine MANOMBA BOULINGUI, Bridy MOUTOMBI DITOMBI, Philomène KOUNA NDOUONGO, Marielle Karine BOUYOU AKOTET

**Affiliations:** 1 Service d'infectiologie, Centre hospitalier universitaire de Libreville, Gabon; 2 Département de médecine et spécialités médicales, Université des sciences de la santé, BP 4000, Gabon; 3 Département des sciences fondamentales et mixtes, Faculté de médecine, Université des sciences de la santé, BP 4000, Gabon Auteur

**Keywords:** VIH, Délai d'entrée aux soins, Antirétroviraux, Libreville, Gabon, Afrique subsaharienne, HIV, Time to care, Antiretrovirals, Libreville, Gabon, Sub-Saharan Africa

## Abstract

**Introduction:**

Le retard de délai d'entrée aux soins est un obstacle à la mise en route immédiate du traitement antirétroviral (TARV) dès le diagnostic, tel que recommandé par l’Organisation mondiale de la santé. Cette étude a pour but de déterminer et de comparer les délais d'entrée aux soins et les facteurs associés chez des personnes vivant avec le VIH (PvVIH) vues dans deux centres de traitement ambulatoires de Libreville entre 2012 et 2020.

**Matériel et méthodes:**

Étude rétrospective réalisée à partir des dossiers colligés de PvVIH, de janvier 2012 à mars 2020, au sein des deux plus grands Centres de traitement ambulatoire (CTA) de Libreville, celui du Centre hospitalier universitaire de Libreville (CHUL) et celui de l'hôpital de Nkembo. L'entrée aux soins était définie comme précoce lorsque le délai entre le diagnostic de l'infection à VIH et la première consultation au CTA était de moins de 28 jours. Elle était tardive lorsque ce délai était de plus de trois mois. Pour l'analyse, les patients ont été répartis entre deux périodes : 2012-2015 quand le démarrage du traitement était lié au taux de CD4, et 2016-2020, période au cours de laquelle le *Test and Treat* a été adopté au Gabon.

**Résultats:**

Au total, 979 patients ont été nouvellement pris en charge dans les deux CTA et les dossiers de 672 personnes ont pu être exploités. Dans 48,3 % des cas, le diagnostic d'infection à VIH était réalisé à un stade tardif (OMS 3 ou 4). Le délai médian d'entrée aux soins était de 1,2 [IQ : 0-3] mois après le diagnostic de l'infection à VIH. Entre 2016 et 2020, 47 % entraient aux soins dans un délai de moins de 28 jours alors que cette proportion était de 35,7 % en 2012-2015 (p < 0,01). Le pourcentage de PvVIH ayant eu une entrée aux soins tardive était comparable entre les deux périodes (14,4 % vs 15,9 % en 2012-2015; p = 0,62). Les facteurs associés à un délai tardif étaient le stade OMS 3, la non-réalisation du dosage des CD4, la profession et la grossesse (p<0,05).

**Conclusion:**

Le délai d'entrée aux soins est encore long à l'ère du *Test and Treat* à Libreville. Une meilleure connaissance des facteurs associés et une approche décentralisée et intégrée de la prise en charge de l'infection à VIH permettraient d'atteindre le 2^e^ pilier des « 95-95-95 » à Libreville.

## Abstract (en)

**Introduction:**

Delays in entry to care are a barrier to immediate initiation of antiretroviral therapy (ART) at diagnosis, as recommended by the World Health Organization. The aim of this study was to determine and compare delays in entry into care and associated factors among people living with HIV (PLHIV) seen at two outpatient treatment centers in Libreville between 2012 and 2020.

**Materials and methods:**

Retrospective study based on PLHIV records collected from January 2012 to March 2020 at the two largest outpatient treatment centers (CTA) in Libreville, that of the Centre Hospitalier Universitaire de Libreville (CHUL) and that of Nkembo Hospital. Early entry into care was defined as less than 28 days between diagnosis of HIV infection and first consultation at the CTA. Late entry was defined as more than three months. For analysis, patients were divided into two periods: 2012-2015, when treatment initiation was linked to CD4 count, and 2016-2020, the period when the *Test and Treat* method was introduced in Gabon.

**Results:**

A total of 979 patients were newly treated in the two CTAs, and the records of 672 individuals could be used. In 48.3% of the cases, HIV infection was diagnosed at a late stage (WHO 3 or 4). The median time to entry into care was 1.2 [IQ: 0-3] months after diagnosis of HIV infection. Between 2016 and 2020, 47% entered care in less than 28 days, compared with 35.7% in 2012-2015 (p < 0.01). The percentage of PLHIV with late entry into care was comparable between the two periods (14.4% vs. 15.9% in 2012-2015; p = 0.62). Factors associated with late entry were WHO stage 3, failure to achieve CD4 count, employment, and pregnancy (p<0.05).

**Conclusion:**

In the era of *Test and Treat* in Libreville, the delay in seeking care is still long. A better understanding of the associated factors and a decentralized, integrated approach to the management of HIV infection would make it possible to achieve the second pillar of “95-95-95” target in Libreville.

## Introduction

L'initiation rapide du traitement antirétroviral (TARV) permet de stopper la progression de l'infection à VIH vers le stade sida et de limiter la transmission du virus. A l’ère de l'accès gratuit aux TARV efficaces et au *Test and Treat*, les patients vivants avec le VIH (PvVIH) ont la possibilité d’être pris en charge précocement et d'avoir un meilleur pronostic [14, 16, 34]. Une mise en route immédiate du suivi et des soins adaptés au moment du diagnostic de l'infection à VIH constituent des éléments clés du succès vers les « 95-95-95 » tels que recommandés par l’Organisation mondiale de la santé (OMS). L'objectif est que 95 % des PvVIH connaissent leur statut sérologique, que 95 % bénéficient d'un TARV et que 95 % des personnes sous TARV aient une charge virale indétectable [21]. L'accès aux soins des PvVIH nouvellement diagnostiqués est défini comme la réalisation du premier rendez-vous ambulatoire du patient avec un prestataire de soins qui possède les compétences et la capacité de traiter l'infection et de prescrire un TARV. Dans certains pays, il est recommandé que ce premier contact entre le PvVIH nouvellement diagnostiqué et la structure de prise en charge spécialisée de l'infection à VIH se fasse dans un délai maximum de 30 jours suivant le diagnostic [27]. Le retard d'entrée aux soins et donc d'accès au TARV est à l'origine d'une progression plus rapide de la maladie, d'une mortalité importante et d'un risque plus élevé de transmission du virus [11, 19]. En 2015, l’OMS a publié des lignes directives sur le dépistage du VIH, en recommandant de réduire le délai entre le diagnostic de l'infection et le démarrage du TARV à 7 jours [23]. Les recommandations pour la mise en route du TARV ont également évolué passant d'un taux de CD4 < 200/mm^3^ à un taux < 500/mm^3^ en 2013, puis à une initiation du traitement dès le diagnostic, quel que soit le taux de CD4 en 2016 [5, 29, 33].

Au Gabon en 2020, 73 % des PvVIH connaissaient leur statut sérologique, mais seulement 54 % d'entre eux avaient accès à une trithérapie antirétrovirale [23]. Des études menées il y a plus de 12 ans rapportaient de longs délais d'entrée aux soins dans cette population. Dans plus de 60 % des cas, les patients débutaient leur suivi au stade 3 ou 4 de la maladie ou avec un taux de CD4 < 200/mm^3^, alors que depuis 2013, une entrée aux soins dans les 7 à 30 jours suivant le diagnostic est préconisée [19, 20]. Suite à l'adoption des nouvelles directives de prise en charge de l'infection à VIH, plusieurs sites de dépistage ont ouvert dans toutes les provinces du pays. Le diagnostic du VIH est effectué systématiquement en consultation prénatale et proposé aux adultes dans la majorité des Centres hospitaliers universitaires. Le délai d'entrée aux soins et celui d'initiation du TARV sont des indicateurs de la qualité des programmes de lutte. Plus leur accessibilité et leur fréquentation sont facilitées et précoces, plus grande est la probabilité d'avoir un meilleur suivi chez la majorité des PvVIH et donc d'atteindre l'objectif des « 95-95-95 ».

Notre étude a pour objectif d'analyser les délais d'entrée aux soins avant et après l'adoption du *Test and Treat* et d'identifier des facteurs associés dans une population de PvVIH suivie dans les deux principaux CTA de Libreville. Plus de 60 % des personnes infectées au Gabon vivent dans cette agglomération.

## Patients et méthodes

Cette étude rétrospective a été menée au sein des deux plus grands CTA de prise en charge des PvVIH, celui du Centre hospitalier universitaire de Libreville (CHUL) et celui de l'hôpital de Nkembo. Elle a porté sur les dossiers colligés de patients qui comportaient la date de diagnostic de confirmation de l'infection à VIH, celle de la première consultation dans les CTA et leur numéro de téléphone. Seules les données de ceux qui ont pu être contactés et qui ont donné leur consentement oral pour l'exploitation de leurs informations ont été retenues pour l'analyse. Ces informations ont été complétées par celles contenues dans les différents registres des deux CTA. Les variables suivantes ont été rapportées sur une fiche de recueil de données :

- la date du diagnostic de l'infection à VIH,
- la date de la première consultation dans le CTA,
- les caractéristiques sociodémographiques : date de naissance, genre, profession,
- les caractéristiques cliniques : circonstances du diagnostic de l'infection à VIH, le stade clinique (stade OMS) à la première consultation,
- l'existence d'un dosage des CD4 à l'entrée aux soins.

En tenant compte du délai habituel de deux à quatre semaines compris entre le diagnostic, l'envoi du résultat au médecin traitant, la prise de rendez-vous dans le CTA et l'entrée dans la file active des nouveaux PvVIH à Libreville, et en considérant les recommandations du *Centers for Disease Control*, l'entrée aux soins était définie comme précoce lorsque le délai entre le diagnostic et la première consultation au CTA était de moins de 28 jours [31]. Elle était tardive lorsque le délai était de plus de trois mois.

Pour l'analyse comparative des délais d'accès aux soins, les patients ont été répartis en fonction de deux périodes : 2012-2015 quand la recommandation de mise sous TARV était un taux de CD4 inférieur à 200 puis 500/mm^3^ et 2016-2020, période au cours de laquelle le *Test and Treat* a été adopté au Gabon.

L’étude a été approuvée par le comité d’éthique pour la recherche sous le n° : 027/2022/CNE/P. Pour chaque patient inclus, un numéro d'identifiant unique remplaçait le nom et le prénom. Une double saisie des données a été effectuée sur un fichier Excel. Elles ont été analysées à l'aide du logiciel Statview 5.0. Les variables quantitatives ont été comparées grâce aux tests de Khi^2^ et Fisher exact. Les variables qualitatives exprimées en médiane [25^e^-75^e^ percentiles] ou en moyenne (± déviation standard), ont été comparées à l'aide des tests Mann-Whitney et Kruskal-Wallis. Le seuil de significativité était de 0,05 (p<0,05).

## Résultats

Parmi les 979 dossiers sélectionnés, ceux de 672 PvVIH (353 en 2012-2015 et 319 en 2016-2020) ayant accepté de participer à l’étude comportaient à la fois la date du diagnostic de l'infection à VIH et la date de leur première visite de suivi. Le genre était connu pour 669 d'entre eux avec 70,7 % (n=473) de femmes. Plus des trois quarts (77,5 %; n = 521/672) étaient âgés de 25 à 54 ans, l’âge médian étant de 42 (34-51) ans. Les circonstances du diagnostic du VIH n’étaient connues que pour 499 patients : dépistage volontaire (n = 213/499; 42,7%) ou prénatal (n = 81/499; 16,2 %), existence de maladies opportunistes (n = 126/499; 25,3 %), suspicion clinique (n = 64; 12,8 %), dépistage du partenaire d'un PvVIH (n = 15; 3 %).

Les patients des deux périodes étaient comparables en ce qui concerne les variables sociodémographiques et les circonstances de diagnostic du VIH (Tableau I).

**Tableau I:** Caractéristiques de la population

	2012-2015		2016-2018		p
	N	%	N	%	
**Sexe**					**0,41**
hommes	106	30,2	90	28,3	
femmes	245	69,8	228	71,7	
Tranches d’âge					**0,53**
< 25 ans	8	2,2	10	3,1	
25-54 ans	269	76,2	252	79,0	
> 54 ans	76	21,5	57	17,9	
**Catégorie socioprofessionnelle**					**0,75**
cadre	23	7,9	24	8,5	
ouvrier	104	35,7	92	32,7	
sans emploi	164	56,3	165	58,7	
Circonstance du diagnostic					**0,09**
dépistage volontaire	99	37,5	114	48,5	
bilan du conjoint	8	3,0	7	3,0	
bilan prénatal	45	17,0	36	15,3	
suspicion clinique	41	15,5	23	9,8	
infection opportuniste	71	27,0	55	23,4	

Globalement, 580 (86,3 %) des patients ont eu un dosage de CD4 à l'entrée aux soins sans différence significative entre les deux périodes (87,3 % entre 2012 et 2015 et 85,3 % à partir de 2016) (p = 0,46). Le stade OMS était connu pour 658 patients, 318 (48,3 %) étaient aux stades 3-4. Entre 2012 et 2015, les participants étaient aux stades 3 et 4 dans respectivement 24,1 % des cas (n = 83/344) et 22,6 % (n = 78/344); cette proportion était de 27,1 % (n = 8 5/314) et 23,0 % (n = 72/314) entre 2016 et 2020 (p = 0,77) (Tableau I).

**Figure 1:**
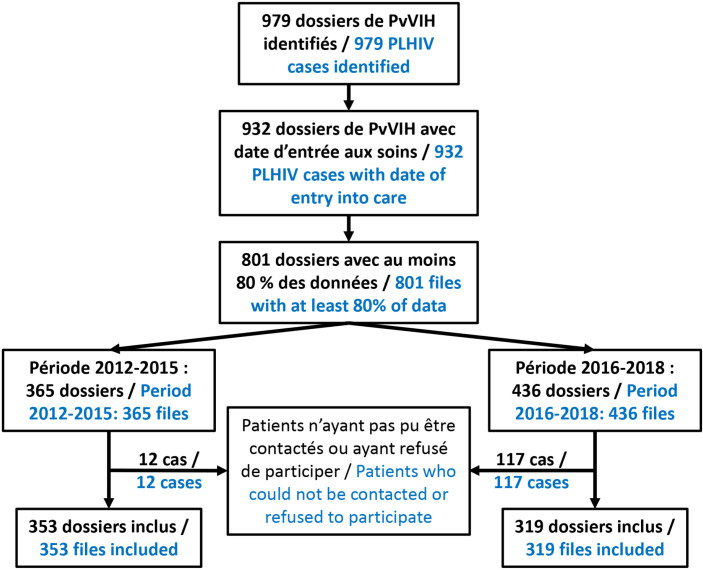
Diagramme de flux

**Figure 2:**
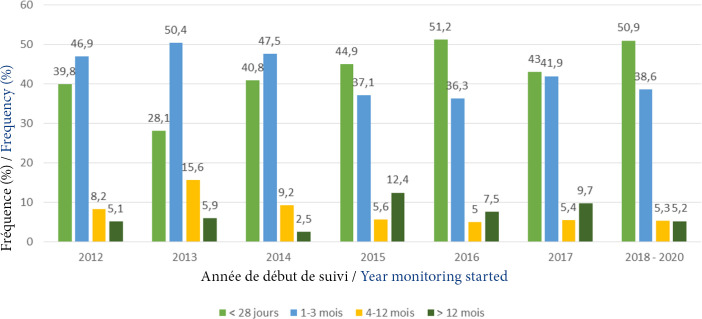
Délai d'entrée aux soins de 2012 à 2020

La proportion des patients ayant un délai d'entrée précoce (< 28 jours après le diagnostic) a significativement augmenté entre 2013 et 2020. Celle des patients vus entre le 4^e^ et le 12^e^ mois a significativement baissé jusqu'en 2016, avant de se stabiliser autour de 5 % (p<0,01) (Fig. 1). De 2012 à 2015, la proportion annuelle des patients ayant débuté leur prise en charge précocement (< 28 jours) est restée inférieure à 45 %. Elle était supérieure à 50 % en 2018-2020 (Fig. 2).

Selon les périodes, 35,7 % (n = 126) des patients avaient consulté dans un délai de moins de 28 jours entre 2012-2015 et 47 % (n = 150) de 2016 à 2020 (p<0,01). La proportion de ceux ayant eu un délai d'accès aux soins de 1 à 3 mois était plus élevée en 2012-2015 (48,4 %; n = 171) par rapport à 2016-2020 (38,6 %; n = 123) (p = 0,01). Une tendance à un délai plus long a été observée chez les patients ayant des symptômes cliniques de la maladie à VIH (p = 0,06).

La proportion de PvVIH ayant eu un délai tardif (≥12 mois) était comparable entre les deux périodes (15,9 % entre 2012-2015 et 14,4 % entre 2016-2020) (p = 0,67).

Le pourcentage de PvVIH ayant eu une entrée aux soins précoce était plus élevé de 2016 à 2020 par rapport à la période de 2012 à 2015 chez les femmes (p = 0,02), chez les moins de 55 ans (p = 0,02), les ouvriers, les personnes se faisant dépister volontairement (p<0,01), celles ayant une maladie opportuniste (p < 0,01) et celles au stade clinique 4 de l’OMS (p = 0,02) (Tableau II). En 2016-2020, il y avait plus fréquemment de PvVIH qui intégraient les files actives précocement par rapport à 2012-2015. Les patients débutant tardivement leur suivi avaient moins fréquemment bénéficié d'un dosage des CD4 au cours de la première visite, notamment en 2016-2020 (60,9 % vs 76,8 % en 2012-2015) (p = 0,08). Cependant, chez les ouvriers et les sans-emplois, cette tendance n’était pas observée (Tableau II). Le pourcentage de patients entrant aux soins plus de trois mois après le diagnostic de l'infection à VIH était comparable entre 2012 et 2020 chez les hommes et les femmes, en fonction des tranches d’âge et dans le groupe des patients aux stades 1, 2 et 3 de l’OMS. Elle a augmenté chez les cadres, les femmes enceintes (p = 0,05), ceux ayant une suspicion clinique de l'infection à VIH (p = 0,04) et ceux débutant leur suivi au stade OMS 4 (p = 0,03). La fréquence d'entrée aux soins entre 1 et 3 mois avait tendance à diminuer en 2016-2020 dans la majorité des groupes, à l'exception des ouvriers, des sans-emplois et de ceux aux stades cliniques 1 et 2, dont les proportions étaient similaires entre les deux périodes (Tableau II).

**Tableau II:** Relation entre les caractéristiques sociodémographiques, les circonstances du diagnostic, le stade clinique OMS, et le délai d'entrée aux soins entre 2012 et 2018

	< 28 jours		1-3 mois		Tardif		p
	2012-2015	2016-2020	2012-2015	2016-2020	2012-2015	2016-2020	
	n (%)		n (%)		n (%)		
**Sexe**							
femmes	87 (35,5)	108 (47,3)	117 (47,6)	85 (37,3)	41 (16,7)	35 (15,4)	0,03
hommes	37 (34,3)	42 (46,7)	54 (50,9)	37 (41,1)	15 (14,5)	11 (12,2)	0,21
**Tranche d’âge**							
< 25 ans	2 (25,0)	4 (40)	6 (75)	6 (60)	0	0	
25-54 ans	98 (36,4)	121 (48)	128 (47,6)	94 (37,3)	43 (16,0)	37 (14,7)	0,02
> 54 ans	26 (34,2)	25 (43,9)	37 (48,7)	23 (40,3)	13 (17,1)	9 (15,8)	0,52
**Catégorie socioprofessionnelle**							
cadre supérieur/moyen	13 (56,5)	14 (58,3)	9 (39,1)	5 (20,8)	1 (4,3)	5 (20,8)	0,13
ouvrier/autres	35 (33,7)	44 (47,8)	48 (46,3)	37 (40,2)	21 (20,2)	11 (12,0)	0,09
sans emploi	57 (34,8)	74 (44,8)	81 (49,4)	69 (41,8)	26 (15,8)	22 (13,3)	0,17
**Circonstance du diagnostic**							
dépistage volontaire	25 (25,2)	53 (46,5)	58 (58,6)	51 (44,7)	16 (16,2)	10 (8,8)	<0,01
bilan prénatal	20 (44,4)	14 (38,9)	20 (44,4)	12 (33,3)	5 (11,1)	10 (27,8)	0,15
infection opportuniste	24 (33,8)	33 (60,0)	38 (53,5)	17 (30,9)	9 (12,7)	5 (9,1)	0,01
suspicion clinique	22 (53,7)	7 (30,4)	15 (36,6)	10 (43,5)	4 (9,8)	6 (26,1)	0,11
**Stade OMS**							
OMS 1+2	63 (34,4)	68 (43,3)	88 (48,1)	63 (40,1)	32 (17,8)	26 (16,6)	0,22
OMS 3	28 (33,7)	37 (43,5)	45 (54,2)	34 (40)	10 (12)	14 (16,5)	0,18
OMS 4	30 (38,5)	43 (59,7)	35 (44,9)	25 (34,7)	13 (16,7)	4 (5,6)	0,01
CD4 à l'entrée aux soins	109(33,4)	134 (49,3)	156 (50,0)	110 (40,4)	43 (14)	28 (10,3)	0,13

## Discussion

Plus de cinq décennies après les premiers cas de VIH, l’épidémie n'est pas contrôlée au niveau mondial. De nombreuses personnes vivant avec le VIH méconnaissent leur statut, sont dépistées tardivement avec comme conséquence, une initiation tardive du TARV. Ce constat est accru en Afrique subsaharienne où on estime que moins de 50 % des PvVIH sont informées de leur statut [25]. Au Gabon en 2023, 76 % des PvVIH connaîtraient leur statut [24].

L'entrée aux soins des PvVIH nouvellement diagnostiqués constitue une étape essentielle pour une prise en charge correcte de ces patients, leur garantissant un accès rapide au TARV et des chances d'une suppression rapide de la charge virale. Plus cette intégration dans le *continuum* des soins est précoce, moins la probabilité d'opportunités manquées de mise en route du TARV est grande [3]. Alors que tous les pays d’Afrique subsaharienne ont adopté les « 95-95-95 », il existe très peu de recommandations sur le délai maximum d'entrée aux soins et sur les interventions permettant de garantir un accès rapide et une rétention des PvVIH dans le *continuum* des soins. En 2015, l’Association internationale des médecins spécialistes des soins du sida a inclus dans ses directives pour l'amélioration du parcours de soins des PvVIH, de référer rapidement celles qui sont nouvellement diagnostiquées et de sensibiliser celles qui n'ont pas initié leur suivi dans le mois suivant le diagnostic [9]. Les pays d’Amérique du Nord ont fixé ce délai à 30 jours [2, 27]. Au Gabon, seuls 54 % des PvVIH connaissant leur statut étaient sous TARV, suggérant donc des difficultés d'entrée immédiate aux soins [23]. La majorité des participants à la présente étude était des adultes appartenant à la tranche d’âge comprise entre 25 et 54 ans. Cette population est plus exposée à l'infection à VIH.

Au cours des deux périodes, les femmes prédominaient (69,5 % de 2012 à 2015 et 71,7 % de 2016 à 2020), comme rapporté par d'autres auteurs au Cameroun, en Côte d’Ivoire et généralement en Afrique subsaharienne [6, 7, 8, 10]. Cette forte proportion peut s'expliquer par la plus grande fréquentation des centres de dépistage et de prise en charge par les femmes et par les programmes de Prévention de la transmission mère enfant (PTME). L'introduction du dépistage de l'infection à VIH dans le bilan prénatal ainsi que la stratégie de mise à disposition des ARV dans les centres de suivi prénatal permettent une prise en charge plus rapide des femmes enceintes PvVIH. Ces stratégies facilitent leur identification et leur adhésion rapide aux soins. À l’échelle mondiale, depuis 2015, plus de 50 % des PvVIH sont des femmes ou des jeunes filles. Elles représentaient 47 % en 2019 et 49 % des personnes nouvellement infectées en 2021 [22, 23, 24]. En Afrique subsaharienne les femmes et les jeunes filles représentaient 62,3 % des nouvelles infections en 2023. Cette proportion était de 71 % en 2014 [22, 24]. Les hommes refusent ou retardent le moment du dépistage, et lorsqu'ils sont testés positifs, ont tendance à retarder le début de la prise en charge (données du service d'infectiologies du CHUL). Au début des années 2000, la prévalence des nouvelles infections à VIH chez les jeunes filles adolescentes était deux fois plus élevée (0,8 %) que celle des jeunes garçons (0,4 %) [28]. Alors que 80 % des femmes PvVIH de plus de 15 ans ont accès au TARV, seuls 70 % des hommes de la même tranche d’âge sont concernés [24]. Plus de 8 ans après l'adoption du *Test and Treat* et du déploiement des centres de prise en charge au Gabon, les femmes représentent 53 % des PvVIH, et 44 % des nouvelles infections [26]. Le retard de diagnostic de l'infection à VIH constitue toujours un obstacle à la réduction de la morbidité et de la mortalité attribuables à cette pandémie. Cette situation est à l'origine d'une prise en charge tardive et souvent peu efficace, malgré la disponibilité et une meilleure accessibilité à la trithérapie et au dosage du taux de CD4. Ce dosage reste le principal marqueur utilisé pour le suivi biologique des patients (80 %); il est souvent utilisé comme indicateur d'entrée aux soins dans les pays où son accès est facilité pour les patients [2, 9]. Ce retard au diagnostic et à l'initiation du suivi concerne près de la moitié (48,3 %) des patients suivis à Libreville. Ceux-ci sont souvent à un stade avancé de l'infection à VIH (stades OMS 3 et 4) au Gabon et au Sénégal (64,5 %) [15, 17]. En moyenne, 50 % des PvVIH entrent dans les files actives à un stade avancé de la maladie. Le dépistage, souvent réalisé devant une infection opportuniste, expliquerait la fréquence élevée d'entrée tardive aux soins. Ces personnes représentent des occasions manquées de prise en charge efficace malgré les approches récentes de dépistage et de traitement précoce. La présence d'infections opportunistes évolutives et l'altération importante de leur état général à l'entrée aux soins, ou les ruptures d'approvisionnement en ARV, ont pour conséquence majeure l'allongement du délai de mise sous trithérapie [12]. Ces résultats montrent que l'atteinte des « 95-95-95 » ne dépend pas uniquement de la disponibilité des CTA et du TARV. Une approche intégrée standardisée, associant l'intensification de la sensibilisation de la population sur la nécessité de connaître son statut VIH chez toute personne en activité sexuelle, sur l'importance de débuter précocement le TARV, sur l'efficacité de ce traitement, sur le danger du déni et la lutte contre la stigmatisation et l'exclusion, est nécessaire.

L'accès immédiat aux soins et au TARV réduit considérablement la morbidité liée au VIH et améliore la survie des PvVIH [4]. Dans l’étude de Lanoy *et al.*, le taux de létalité à 6 mois des patients ayant eu un accès retardé aux soins était de 4,9 % contre 0,3 % pour les patients pris en charge immédiatement. Cette surmortalité restait significative jusqu’à quatre ans après le début de leur prise en charge [13]. Plusieurs facteurs sont associés au retard d'entrée aux soins, parmi lesquels l'absence de décentralisation de la prise en charge des PvVIH, le déni, la stigmatisation, les conditions socio-économiques, les difficultés d'accès aux soins, l'organisation du système des soins, le manque de communication et de sensibilisation des patients [1]. Au Gabon, le diagnostic de l'infection à VIH est réalisé dans la majorité des structures de santé et dans les laboratoires publics et privés, alors que la prise en charge reste centralisée dans des centres ou services dédiés qui sont souvent éloignés des sites de dépistage. Bien que les patients soient référés dans ces sites après le diagnostic, l'absence de dossiers médicaux électroniques et donc de partage d'informations entre les prestataires de soins, ne permet pas toujours de vérifier le contact effectif entre le patient et le service de prise en charge. Par ailleurs, il n'existe pas de recommandation sur le délai rapide d'entrée aux soins et sur les modalités pratiques individuelles et organisationnelles nécessaires à une prise en charge rapide. Malgré l'absence de données sur le devenir des participants à la présente étude, les résultats obtenus permettent déjà d'alerter les autorités de santé et le Programme national de lutte contre le sida sur la nécessité d'intensifier la communication et la sensibilisation sur l'intérêt d'un dépistage précoce de l'infection à VIH et d'un délai court d'entrée aux soins. Cet engagement rapide dans le suivi de la maladie passe par une approche décentralisée et intégrée du VIH/sida à la prise en charge des pathologies chroniques dans les structures sanitaires publiques et privées. De plus, une étude sur l'itinéraire thérapeutique des PvVIH ayant une entrée aux soins de plus de 15 jours, permettra de compléter les résultats de ce travail.

## Conclusion

Le délai d'entrée aux soins des PvVIH est tardif pour la majorité de ceux qui consultent à Libreville. Ces occasions manquées de prise en charge précoce doivent inciter à repenser les stratégies de communication, de sensibilisation des populations, associées à un modèle différencié du *continuum* des soins pour atteindre l'objectif des « 95-95-95 » au Gabon.

## Autorisation administrative

027/2022/CNE/SG/P

## Sources de financement

EDCTP CSA2020 CANTAM 3 Epi PROJECT

## Contribution des auteurs

Michèle Marion NTSAME OWONO : revue de littérature, rédaction du manuscrit.

Magalie ESSOMEYO NGUE MEBALE, Charleine MANOMBA BOULINGUI, Bridy MOUTOMBI DITOMBI, Philomène KOUNA NDOUONGO, Marielle Karine BOUYOU AKOTET : apport critique, correction du manuscrit et approbation de la version finale à publier.

## Déclaration de liens d'intérêts

Les auteurs déclarent ne pas avoir de liens d'intérêt.
