# Supplementary material for: Time of entry into care of people living with HIV in two outpatient treatment centers of Libreville, Gabon, between 2012 and 2020
Source: Med Trop Sante Int. 2025 Feb 11;5(1):mtsi.v5i1.2025.537. [Article in French] doi: 10.48327/mtsi.v5i1.2025.537 (PMC12001990; doi:10.48327/mtsi.v5i1.2025.537)
Supplement: Related texts

# Research article: Time of entry into care of people living with HIV in two outpatient treatment centers of Libreville, Gabon, between 2012 and 2020

## Introduction

Rapid initiation of antiretroviral therapy (ART) can halt the progression of HIV infection to AIDS and limit viral transmission. In the era of free access to effective ART and *Test and Treat*, people living with HIV (PLHIV) have the opportunity to be treated early and have a better prognosis [14, 16, 34]. Immediate initiation of follow-up and appropriate care at the time of diagnosis of HIV infection is key to achieving the World Health Organization (WHO) recommended ‘95-95-95’ target. The goal is for 95% of PLHIV to know their serostatus, for 95% to benefit from ART and for 95% of those on ART to have an undetectable viral load [21]. Access to care for newly diagnosed PLHIV is defined as completion of the patient's first outpatient appointment with a health care provider who has the skills and capacity to treat the infection and prescribe ART. In some countries, it is recommended that this first contact between newly diagnosed PLHIV and the specialized HIV care facility should occur within a maximum of 30 days of diagnosis [27]. Delayed entry into care, and therefore access to ART, is the cause of more rapid disease progression, high mortality and increased risk of viral transmission [11, 19]. In 2015, WHO published guidelines on HIV testing, recommending that the time between diagnosis of infection and initiation of ART be reduced to 7 days [23]. Recommendations for ART initiation have also evolved, from a CD4 count < 200/mm^3^ to < 500/mm^3^ in 2013, and then to treatment initiation at diagnosis regardless of CD4 count in 2016 [5, 29, 33].

In Gabon in 2020, 73% of PLHIV knew their serostatus, but only 54% had access to triple antiretroviral therapy [23]. Studies conducted over 12 years ago reported long delays in access to care in this population. In more than 60% of cases, patients started treatment at stage 3 or 4 of the disease or with a CD4 count < 200/mm^3^, whereas since 2013 it is recommended to start treatment within 7 to 30 days of diagnosis [19, 20]. Following the adoption of new guidelines for the management of HIV infection, several screening sites have been opened in all provinces of the country. HIV diagnosis is routinely offered at antenatal clinics and to adults at most university hospitals. The time taken to access care and initiate ART is an indicator of the quality of control programmes. The easier and earlier they are to access and use, the greater the likelihood of better follow-up of the majority of PLHIV and thus of achieving the “95-95-95” target.

The aim of our study is to analyze delays in entry into care before and after the introduction of the *Test and Treat* method and to identify factors associated with these delays in a population of PLHIV followed up in the two main CTAs in Libreville. More than 60% of infected people in Gabon live in this urban area.

## Patients and methods

This retrospective study was conducted in the two largest CTAs caring for PLHIV, the Centre Hospitalier Universitaire de Libreville (CHUL) and the Hôpital de Nkembo. Patient records were collected, including the date of diagnosis and confirmation of HIV infection, the date of first consultation at the CTAs, and the patient's telephone number. Only data from patients who could be contacted and who gave verbal consent for their information to be used were included in the analysis. This information was supplemented by the information contained in the various registers of the two CTAs.

The following variables were reported on a data collection form:

- Date of diagnosis of HIV infection,
- Date of first consultation at the CTA,
- Sociodemographic characteristics: date of birth, gender, occupation,
- Clinical characteristics: circumstances of diagnosis of HIV infection, clinical stage (WHO stage) at first consultation,
- CD4 count at admission.

Taking into account the usual delay of 2-4 weeks between diagnosis, transmission of results to the treating physician, booking of an appointment at the CTA and entry into the active file of new PLHIV in Libreville, and considering the recommendations of the Centers for Disease Control, early entry into care was defined as a delay of less than 28 days between diagnosis and the first consultation at the CTA [31]. Late entry was defined as more than three months.

For the comparative analysis of delays in entry into care, patients were divided into two time periods: 2012-2015, when the recommendation to start ART was a CD4 count below 200 then 500/mm^3^, and 2016-2020, the period during which *Test and Treat* was introduced in Gabon.

The study was approved by the Research Ethics Committee under no: 027/2022/CNE/P. For each patient included, a unique identification number replaced the surname and first name.

Data were entered twice into an Excel file. They were analyzed using Statview 5.0 software. Quantitative variables were compared using Chi^2^ and Fisher exact tests. Qualitative variables, expressed as median [25^th^-75^th^ percentile] or mean (± standard deviation), were compared using the Mann-Whitney and Kruskal-Wallis tests. The significance level was set at 0.05 (p<0.05).

## Results

Of the 979 records selected, those of 672 PLHIV (353 in 2012-2015 and 319 in 2016-2020) who agreed to participate in the study included both the date of diagnosis of HIV infection and the date of their first follow-up visit. Gender was known for 669 of them, with 70.7% (n = 473) being women. More than three quarters (77.5%; n = 521/672) were aged between 25 and 54 years, with a median age of 42 years (34-51). The circumstances of HIV diagnosis were known for only 499 patients: voluntary (n = 213/499; 42.7%) or prenatal (n = 81/499; 16.2%) screening, presence of opportunistic diseases (n = 126/499; 25.3%), clinical suspicion (n = 64; 12.8%), screening of the partner of a PLHIV (n = 15; 3%).

Patients in both periods were comparable with respect to sociodemographic variables and circumstances of HIV diagnosis (Table I).

**Table I:** Patients characteristics

	2012-2015		2016-2018		p
	N	%	N	%	
**Gender**					**0,41**
male	106	30,2	90	28,3	
female	245	69,8	228	71,7	
Age groups					**0,53**
years	8	2,2	10	3,1	
years	269	76,2	252	79,0	
> 54 years	76	21,5	57	17,9	
**Socioprofessional category**					**0,75**
executive	23	7,9	24	8,5	
manual worker	104	35,7	92	32,7	
unemployed	164	56,3	165	58,7	
Circumstances of diagnosis					**0,09**
voluntary screening	99	37,5	114	48,5	
spouse's assessment	8	3,0	7	3,0	
prenatal assessment	45	17,0	36	15,3	
clinical suspicion	41	15,5	23	9,8	
opportunistic infection	71	27,0	55	23,4	

Overall, 580 (86.3%) patients had a CD4 test at entry into care, with no significant difference between the two periods (87.3% between 2012 and 2015 and 85.3% from 2016 onwards) (p = 0.46). WHO stage was known for 658 patients, 318 (48.3%) were in stages 3-4. Between 2012 and 2015, 24.1% (n = 83/344) and 22.6% (n = 78/344) of participants were in stages 3 and 4, respectively; between 2016 and 2020, this proportion was 27.1% (n = 85/314) and 23.0% (n = 72/314), respectively (p = 0.77) (Table I).

The proportion of patients with an early delay in presentation (< 28 days after diagnosis) increased significantly between 2013 and 2020. The proportion of patients seen between 4 and 12 months decreased significantly until 2016, before stabilizing at around 5% (p<0.01) (Figure 1). From 2012 to 2015, the annual proportion of patients starting treatment early (<28 days) remained below 45%. In 2018-2020, it was over 50% (Fig. 2). Depending on the time period, 35.7% (n = 126) of patients in 2012-2015 and 47% (n = 150) in 2016-2020 had a consultation within 28 days (p<0.01). The proportion of patients with a delay in accessing care of 1-3 months was higher in 2012-2015 (48.4%; n = 171) compared to 2016-2020 (38.6%; n = 123) (p = 0.01). A trend towards longer delays was observed in patients with clinical symptoms of HIV disease (p = 0.06).

**Figure 1:** Flowchart

**Figure 2:** Entry into care data from 2012 to 2020

The proportion of PLHIV with late delay (≥12 months) was comparable between the two periods (15.9% between 2012-2015 and 14.4% between 2016-2020) (p = 0.67).

The proportion of PLHIV with early entry into care was higher in 2016-2020 compared with 2012-2015 among women (p = 0.02), those younger than 55 years (p = 0.02), blue-collar workers, those who tested voluntarily (p<0.01), those with opportunistic diseases (p<0.01), and those with WHO clinical stage 4 (p = 0.02) (Table II). In 2016-2020, there were more PLHIV who entered the active files early than in 2012-2015. Patients who started their follow-up late were less likely to have a CD4 count at the first visit, especially in 2016-2020 (60.9% vs. 76.8% in 2012-2015) (p = 0.08). However, this trend was not observed among blue-collar workers and the unemployed (Table II). Percentage of patients entering care more than three months after diagnosis of HIV infection was comparable between 2012 and 2020 among men and women, according to age groups and in the group of patients in WHO stages 1, 2 and 3. It increased among managers, pregnant women (p = 0.05), those with clinical suspicion of HIV infection (p = 0.04), and those entering care at WHO stage 4 (p = 0.03). The frequency of entry into care between 1 and 3 months tended to decrease between 2016-2020 in most groups, with the exception of blue-collar workers, the unemployed and those in clinical stages 1 and 2, whose proportions were similar between the two periods (Table II).

**Table I:** Relationship between sociodemographic characteristics, circumstances of diagnosis, WHO clinical stage, and time of entry into care between 2012 and 2018

	< 28 days		1-3 months		Delayed		p
	2012-2015	2016-2020	2012-2015	2016-2020	2012-2015	2016-2020	
	n (%)		n (%)		n (%)		
**Gender**							
female	87 (35,5)	108 (47,3)	117 (47,6)	85 (37,3)	41 (16,7)	35 (15,4)	0,03
male	37 (34,3)	42 (46,7)	54 (50,9)	37 (41,1)	15 (14,5)	11 (12,2)	0,21
**Age groups**							
< 25 years	2 (25,0)	4 (40)	6 (75)	6 (60)	0	0	
25-54 years	98 (36,4)	121 (48)	128 (47,6)	94 (37,3)	43 (16,0)	37 (14,7)	0,02
> 54 years	26 (34,2)	25 (43,9)	37 (48,7)	23 (40,3)	13 (17,1)	9 (15,8)	0,52
**Socioprofessional category**							
upper/middle management	13 (56,5)	14 (58,3)	9 (39,1)	5 (20,8)	1 (4,3)	5 (20,8)	0,13
manual worker/others	35 (33,7)	44 (47,8)	48 (46,3)	37 (40,2)	21 (20,2)	11 (12,0)	0,09
unemployed	57 (34,8)	74 (44,8)	81 (49,4)	69 (41,8)	26 (15,8)	22 (13,3)	0,17
**Circumstances of diagnosis**							
voluntary screening	25 (25,2)	53 (46,5)	58 (58,6)	51 (44,7)	16 (16,2)	10 (8,8)	<0,01
prenatal assessment	20 (44,4)	14 (38,9)	20 (44,4)	12 (33,3)	5 (11,1)	10 (27,8)	0,15
opportunistic infection	24 (33,8)	33 (60,0)	38 (53,5)	17 (30,9)	9 (12,7)	5 (9,1)	0,01
clinical suspicion	22 (53,7)	7 (30,4)	15 (36,6)	10 (43,5)	4 (9,8)	6 (26,1)	0,11
**WHO stage**							
WHO 1+2	63 (34,4)	68 (43,3)	88 (48,1)	63 (40,1)	32 (17,8)	26 (16,6)	0,22
WHO 3	28 (33,7)	37 (43,5)	45 (54,2)	34 (40)	10 (12)	14 (16,5)	0,18
WHO 4	30 (38,5)	43 (59,7)	35 (44,9)	25 (34,7)	13 (16,7)	4 (5,6)	0,01
CD on entry into care	109(33,4)	134 (49,3)	156 (50,0)	110 (40,4)	43 (14)	28 (10,3)	0,13

## Discussion

More than five decades after the first cases of HIV, the global epidemic remains uncontrolled. Many people living with HIV are unaware of their status and are tested late, resulting in late initiation of ART. This situation is particularly acute in sub-Saharan Africa, where less than 50% of people living with HIV are estimated to know their status [25]. In Gabon, 76% of PLHIV would know their status by 2023 [24]. Newly-diagnosed PLHIV entry into care is an essential step in the proper management of these patients, ensuring rapid access to ART and the chance of rapid viral load suppression. The earlier this integration into the *continuum* of care is achieved, the less likely there will be missed opportunities to initiate ART [3]. While all sub-Saharan African countries have adopted the “95-95-95”, there are very few recommendations on the maximum time to entry into care and interventions to ensure rapid access and retention of PLHIV in the care *continuum.* In 2015, the International Association of Providers of AIDS Care included in its guidelines for improving access and utilization of HIV services, the rapid referral of newly diagnosed individuals and increased awareness of those who have not initiated follow-up within one month of diagnosis [9]. North American countries have set this deadline at 30 days [2, 27]. In Gabon, only 54% of PLHIV who knew their status were on ART, suggesting difficulties in immediate linkage to care [23].

The majority of participants in the present study were adults aged 25-54 years. This population is more exposed to HIV infection.

In both periods, women predominated (69.5% from 2012 to 2015 and 71.7% from 2016 to 2020), as reported by other authors in Cameroon, Côte d’Ivoire, and in Sub-Saharan Africa in general [6, 7, 8, 10]. This high proportion can be explained by the greater frequency of women's attendance at screening and care centers, as well as prevention of mother-to-child transmission (PMTCT) programs. The introduction of HIV screening as part of antenatal care and the strategy of making ARVs available at antenatal care centers means that HIV-positive pregnant women can be cared for more quickly. These strategies facilitate their identification and rapid engagement in care. Globally, more than 50% of PLHIV have been women or girls since 2015. They will account for 47% of newly infected people in 2019 and 49% in 2021 [22, 23, 24]. In sub-Saharan Africa, women and girls accounted for 62.3% of new infections in 2023. This proportion was 71% in 2014 [22, 24]. Men refuse or delay screening, and if they test positive, they tend to delay starting treatment (data from CHUL Infectious Diseases Department). In the early 2000s, the prevalence of new HIV infections among adolescent girls (0.8%) was twice that of adolescent boys (0.4%) [28]. While 80% of HIV-infected women aged 15 years and older have access to ART, only 70% of men in the same age group do [24]. More than 8 years after the introduction of *Test and Treat* and the establishment of care centers in Gabon, women represent 53% of PLHIV and 44% of new infections [26].

Delayed diagnosis of HIV infection remains an obstacle to reducing the morbidity and mortality of this pandemic. This situation results in late and often ineffective treatment, despite the availability and improved accessibility of triple therapy and CD4 testing. The CD4 count remains the main marker for biological monitoring of patients (80%) and is often used as an indicator for treatment initiation in countries where it is readily available to patients [2, 9]. This delay in diagnosis and initiation of follow-up affects almost half (48.3%) of the patients monitored in Libreville. These patients are often at an advanced stage of HIV infection (WHO stages 3 and 4) in Gabon and Senegal (64.5%) [15, 17]. On average, 50% of people living with HIV enter the active file at an advanced stage of the disease. Screening, often done in the presence of an opportunistic infection, would explain the high frequency of late entry into care. These individuals represent missed opportunities for effective care, despite recent screening and early treatment approaches. The presence of progressive opportunistic infections and a significant deterioration in their general condition on entry into care, or disruptions in ARV supply, have the greatest impact on prolonging the delay in starting triple therapy [12]. These results show that achieving the “95-95-95” target does not depend solely on the availability of ACTs and ART. An integrated, standardized approach is needed that combines increased public awareness of the need for all sexually active people to know their HIV status, the importance of early initiation of ART, the effectiveness of this treatment, the risk of denial, and the fight against stigma and exclusion.

Prompt access to care and ART significantly reduces HIV-related morbidity and improves survival in PLHIV [4]. In the study by Lanoy et al., the 6-month case fatality rate for patients with delayed access to care was 4.9%, compared with 0.3% for patients treated immediately. This excess mortality remained significant up to four years after initiation of care [13]. Several factors are associated with delayed entry into care, including lack of decentralization of care for PLHIV, denial, stigma, socioeconomic conditions, difficulties in accessing care, organization of the care system, and lack of communication and patient awareness [1]. In Gabon, HIV infection is diagnosed in the majority of health facilities, and public and private laboratories, while care remains centralized in specialized centers or services, often far from screening sites. Although patients are referred to these sites after diagnosis, the lack of electronic medical records and therefore of information sharing between service providers means that it is not always possible to verify actual contact between the patient and the service. In addition, there are no recommendations on how quickly a patient should be admitted to care, or on the individual and organizational practicalities required for rapid care. Despite the lack of data on the outcome of participants in the present study, the results already serve to alert health authorities and the National AIDS Control Program to the need to intensify communication and awareness of the benefits of early detection of HIV infection and rapid entry into care. This rapid involvement in disease control requires a decentralized and integrated approach to HIV/AIDS and the management of chronic pathologies in public and private health structures. In addition, a study of the therapeutic pathway of PLHIVwith an entry into care of more than 15 days will complement the results of this work.

## Conclusion

The majority of PLHIV seeking care in Libreville do so late. These missed opportunities for early care should prompt a rethinking of communication and awareness-raising strategies, combined with a differentiated model of the care *continuum*, to achieve the “95-95-95” target in Gabon.

## Administrative approval

027/2022/CNE/SG/P

## Funding

EDCTP CSA2020 CANTAM 3 Epi PROJECT

## Authors’ contributions

Michèle Marion NTSAME OWONO: literature review, drafting of the manuscript.

Magalie ESSOMEYO NGUE MEBALE, Charleine MANOMBA BOULINGUI, Bridy MOUTOMBI DITOMBI, Philomène KOUNA NDOUONGO, Marielle Karine BOUYOU AKOTET: critical input, revision of the manuscript and approval of the final version for publication.

## Declaration of interests

The authors declare no conflict of interests.
